# The Immediate Effects of Deep Pressure on Young People with Autism and Severe Intellectual Difficulties: Demonstrating Individual Differences

**DOI:** 10.1155/2017/7534972

**Published:** 2017-01-09

**Authors:** Lana Bestbier, Tim I. Williams

**Affiliations:** ^1^Priors Court Foundation, Thatcham, UK; ^2^Institute of Education, University of Reading, Reading, UK

## Abstract

**Background:**

Deep pressure is widely used by occupational therapists for people with autism spectrum disorders. There is limited research evaluating deep pressure.

**Objective:**

To evaluate the immediate effects of deep pressure on young people with autism and severe intellectual disabilities.

**Methods:**

Mood and behaviour were rated for 13 pupils with ASD and severe ID before and after deep pressure sessions.

**Results:**

Sufficient data was available from 8 participants to be analysed using Tau-U, a nonparametric technique that allows for serial dependence in data. Six showed benefits statistically. Five of these showed benefits across all domains, and one showed benefits on three out of five domains.

**Relevance to Clinical Practice:**

Deep pressure appears to be of immediate benefit to this population with autism and severe ID, but the heterogeneity of response suggests that careful monitoring of response should be used and deep pressure discontinued when it is no longer of benefit.

**Limitations:**

This is an open label evaluation study using rating scales.

**Recommendations for Future Research:**

Future studies of the use of deep pressure should use physiological response measures, in addition to blinded raters for aspects of behaviours such as attitude to learning psychological health not captured physiologically.

## 1. Introduction

Deep pressure has been defined as the “sensation produced when an individual is hugged, squeezed, stroked, or held” [1]. It is widely used by occupational therapists working with children with autism spectrum disorders (ASD) [2] and is thought to be rewarding, reducing symptoms of stress and anxiety and improving performance in school [3]. Deep pressure is based on sensory integration theory [4] as initially developed by Ayres in the 1960s and 1970s [5]. The use of deep pressure for individuals with autism spectrum disorders has been widely discussed since Temple Grandin described her self-designed machine (hug machine) for giving her the pressure sensations that she craved [6]. Other types of deep pressure therapy include weighted garments, swaddling, holding, stroking, hugging, squeezing, and therapeutic brushing [7].

A number of reviews have concluded that sensory integration has insufficient evidence for therapeutic and educational use unless it is carefully evaluated [2]; however, other reviews have reached different conclusions. Case-Smith et al. [8] helpfully distinguished between sensory integration techniques (SI, “play and sensory-enhanced interactions to elicit the child's adaptive responses”) and sensory-based interventions (SBI, “adult-directed sensory modalities that are applied to the child to improve behaviours associated with modulation disorders”). Deep pressure is generally a technique that is administered by caregivers and so falls into the category of SBI rather than being an implementation of sensory integration.

The evidence for SBIs such as deep pressure is less convincing, in part because of the heterogeneity of interventions and in part because the reviews do not examine the same sets of studies. For instance, Case-Smith et al. [8] identified 2 studies of brushing and joint compression as compared with 7 studies using weighted vests, 2 studies using therapy balls, and 4 studies using multiple methods including deep pressure. Losinski et al. [4] reviewed 14 studies of weighted or snug vests and 2 of Wilbarger brushing. Yunus et al. [9] reviewed 4 studies of weighted vests, three of massage, and one each of brushing, horse riding intervention, and therapy ball chairs.

There seems to be agreement between the systematic reviews that weighted vests do not seem to confer any benefit on participants. However, Chen et al. [7] using a within subjects design (*N* = 12) found that weighted blankets reduced signs of dental anxiety in typically developing students when measured physiologically but not behaviourally. Blairs et al. [10] provided a programme of noncontingent deep pressure using bed linen (swaddling) to an adult with autism, severe anxiety, and intellectual disability. They proposed that he might benefit, because he had previously benefitted from being hugged by his mother when upset and because he chose to wear a tightly fitting coat when he was in anxiety provoking situations. As an adult he was observed to be more relaxed when tightly tucked into bed and when being physically restrained. Following the introduction of noncontingent deep pressure, episodes of restraint and as required medication were used less often to control his behaviour. Physiological indicators of stress (blood pressure, heart rate, and respiration rate) also reduced following the introduction of this form of deep pressure.

Other types of SBI have been less well studied. Losinski et al. [4] concluded that Wilbarger brushing had no effect. A previous systematic review of the Wilbarger brushing studies [11] found four studies (only one of which is reviewed by Losinski et al. [4]) from which they concluded that “emerging evidence from these studies warrants future robust research on this topic.” Similarly, Yunus et al. [9] concluded that tactile based therapies (such as massage or brushing) offered level 1 evidence of efficacy in managing behaviour problems.

There is some evidence of short term beneficial effects of squeezing which was not analysed by Case-Smith et al. [8], Losinski et al. [4], or Yunus et al. [9]. A “squeeze machine” controlled by participants themselves (cf. Grandin's “hug machine”) was found to feel more relaxing than a control treatment, although objective measures showed no difference [1]. Edelson et al. [12] tested deep pressure in the Grandin style squeeze machine using a randomized allocation of 12 young people with autism compared with being placed in the machine but without any pressure. Physiological measures (Galvanic Skin Response, GSR) did not show any difference but a parent rating of anxiety did. A post hoc analysis suggested that those with higher arousal preintervention seemed to benefit more than the lower arousal group, suggesting that there might be individual differences in responses.

McGinnis et al. [13] offer a different perspective on sensory-based interventions. Three children with ASD aged between 2 and 7 years who were receiving ABA therapy in a private clinic were allowed to choose objects (blanket, gym mat, and pillow) which could be used to provide deep pressure. The experimental phase required the young people to choose whether to have free play or deep pressure. Two of the children showed a clear preference for deep pressure over free play. The third's preferences were confounded by ill health during the experimental phase. The authors concluded that deep pressure was rewarding for the children. Unlike previous studies of SBI this study uses a contingent application of SBI dependent on requesting it. They had planned their study to investigate whether deep pressure could be rewarding, since they were concerned that the use of deep pressure to help individuals calm when distressed might inadvertently reward signs of distress.

In summary, the evidence for deep pressure is equivocal [4, 8, 9], but the reviews cited above fail to cite evidence from case studies. Another evidence suggests that there may be idiosyncratic responses as has been highlighted by McGinnis et al. [13] who demonstrated that only some people will find deep pressure rewarding. A further issue is when to provide deep pressure. With the exception of McGinnis et al. [13] all research cited above seems to have used deep pressure as a means of improving mood and adaptability to the environment. As a result it was used noncontingently and regularly. McGinnis et al. [13] provided deep pressure as a form of rewarding stimulation following task completion and point out that the form of deep pressure may be person specific. An alternative would be to use deep pressure at times when the person was distressed or otherwise in a non-optimal state. This study was designed to provide information about the extent of variability of the immediate responses of young people with autism and severe intellectual disability to deep pressure by providing regular access to deep pressure.

## 2. Methods

### 2.1. Research Design

A pretest-posttest design was employed to evaluate the effects of deep pressure sessions on young people with autism spectrum disorder and severe intellectual disabilities. The study was approved by the ethics subcommittee of the trustees of Priors Court Foundation (Education & Care Committee 2012-2013: Minute number 650 dated November 8, 2012). The agreed procedure included requesting consent from the parents of each of the participants.

### 2.2. Participants

The study took place in a residential school for children with autism and severe intellectual disabilities. 13 young people (2 girls) were selected to take part in the study based on behaviours that were deemed by staff to indicate a desire for deep pressure (cf. [10]). The behaviours that were thought to indicate a need for deep pressure included trying to get in very small spaces, placing people's hands upon their heads, head-banging, and stamping feet. The ages of the young people ranged from 7 yrs and 10 mths to 18 yrs and 7 mths.  Table 1 shows further details of the young people who were involved in the study and whose data included information from more than 15 sessions of deep pressure.

### 2.3. Measures

Visual analogue scales [16] are routinely used by the staff in the school to measure mood and activity of the young people. The visual analogue scales are 10 cm lines anchored at each end with the modifiers “not at all” and “very.” All staff are trained during induction to use these scales. Each young person is usually rated on individually designed scales, but for the purposes of this study the same five areas of mood and behaviour were rated by the one of the staff working with the young person: calmness, engagement with activities, responsiveness to instructions or other stimuli in the environment, happiness, and communicativeness (e.g., the two anchor points for the calmness visual analogue scale were “not at all calm” and “very calm”). The measures were designed to be simple and quick to complete and yet provide indicators of possible short term responses to deep pressure. The staff were also encouraged to provide further written information on the reactions of the young people to planned sessions, and if the young people seemed distressed by the procedures or attempted to withdraw, they were allowed to.

### 2.4. Procedure

Three deep pressure techniques were used: brushing, massage, and squeezing. They were delivered in quiet places with minimal distraction by care and education staff who had been trained by an occupational therapist with a sensory integration qualification. Deep pressure sessions between five and fifteen minutes were scheduled to be delivered up to three times per day during school hours. The deep pressure was delivered over a period of three months on weekdays when the young people were in school. Variations in numbers of sessions per child were due to reasons such as the child being absent from school through ill health, school trips, or insufficient trained staff available to carry out sessions. The staff administering the deep pressure rated the young person's behaviour for the half hour before the session and again thirty minutes after the session.

### 2.5. Analysis

Data will be presented for 8 young people for whom there were more than 15 sessions to evaluate. Additional 5 young people had not completed sufficient sessions for reliable statistical analysis. The statistical analysis uses the Tau-U statistic [17] which measures the difference in data range (nonoverlap) between two phases (A and B), while being able to correct for a moving baseline. It is a nonparametric technique, with statistical power of 91% to 95% of linear regression but when data violates the assumptions of parametric data analysis the power of Tau-U can exceed the parametric techniques to 115% [17]. For the analysis reported here the two phases are before and after deep pressure.

## 3. Results

**Figure 1:**
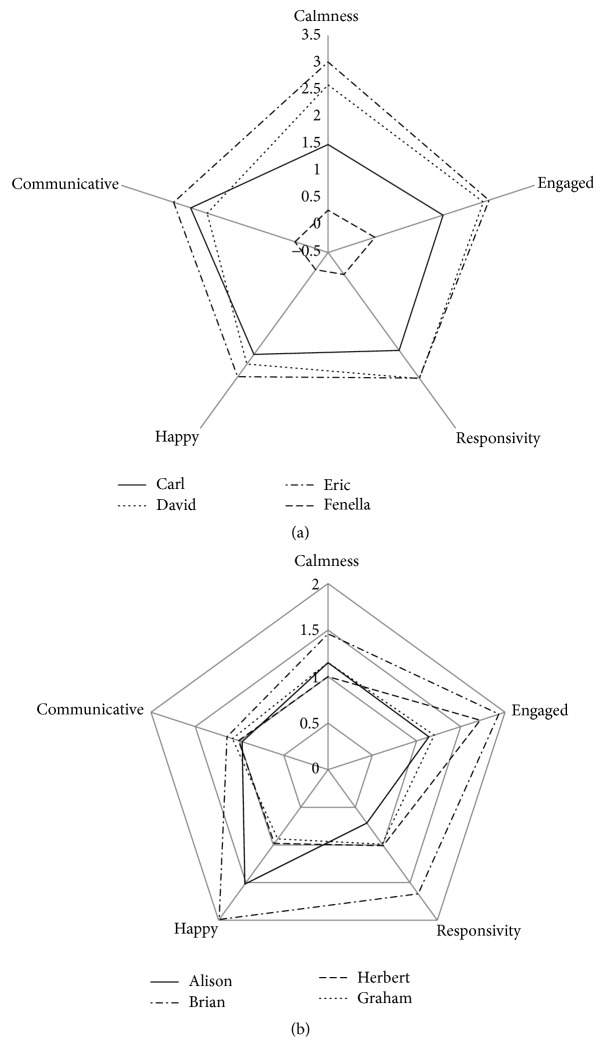
Radar plot of mean change in five rating scales (happy, communicative, calmness, engaged(-ness), and responsivity) for each of the eight participants. The radar plot shows the 5 ratings for each participant, such that the distance from the centre (at which point the change in rating scale = −0.5, i.e., a deterioration) indicates the magnitude of the changes. If every participant made an equal improvement the shapes would be regular pentagons. Rating scales are designed to display positive values for improved functioning in that domain.

Table 2 shows the results as means and standard deviations of the five ratings before and after deep pressure sessions together with associated levels of statistical significance for the changes for each young person aggregated over all of their sessions. Four of the young people showed benefits in all areas measured, two showed no benefits in any area, and further two showed benefits on three and two ratings each.  Figure 1 shows radar plots of the mean change for all five scales and eight participants. The radar plot has five “axes” representing each of the ratings. The five sided shapes (pentagons) represent the mean changes for an individual. It can be seen that Fenella shows the least benefit (the pentagon has short sides). On the other hand Alison shows large consistent benefits across all five ratings. Carl's pentagon is quite irregular which suggests that he benefits in some areas more than others.

## 4. Discussion

Deep pressure appears to be beneficial for most of the young people in this study. The effects were not uniform across all areas of functioning and it is clear that there are individual differences in response to deep pressure that should be taken into account when providing deep pressure interventions. As Figure 1 shows the largest changes across all the participants seem to be in the area of happiness, with possibly the least consistency in calmness. In the course of the study significant numbers of staff rated the behaviour and mood of the young people, which has probably diminished the effect sizes by introducing additional variability in ratings.

The variability of responses to deep pressure may be of importance clinically. Of the eight participants, four (Alison, Brian, Carl, and Eric) showed a wide ranging improvement in rated behaviours. David and Graham's responses to deep pressure were less wide ranging. Both showed improvements in calmness and David was more responsive and engaged. Graham surprisingly showed an increase in communicativeness following sessions of deep pressure suggesting that, for some individuals with autism, communication skills learning may be enabled. Neither Fenella nor Herbert seemed to benefit from deep pressure. It was unclear why this was, but we might note that Fenella differed from the other participants in being a very placid individual who moved around rather slowly. Herbert too was different, in that, left to his own devices, he chose to wrap himself in a blanket suggesting he would prefer a type of swaddling deep pressure rather than the more localized versions being provided in this study. It follows that one question for future research is whether it is possible to predict which individuals will derive benefit from the intervention. There are a number of measures of sensory sensitivity and processing that are believed to predict response to sensory stimulation (cf. [18]) and which could be evaluated.

All but one of the studies so far reported on deep pressure have used the technique noncontingently. For example, Blairs et al. [10] successfully used deep pressure noncontingently as a means to reduce levels of challenging behaviour. However, one of the main claims for the efficacy of deep pressure is that it can be used to improve the mood of the person receiving it when they are already anxious or otherwise upset. Longer term information on the mood and behaviour of people receiving deep pressure could help suggest answers to this question, although it might require further studies using quasi-experimental designs to determine the feasibility of a randomized controlled trial. Consideration should also be given to how deep pressure could be used to improve behaviour, if it is rewarding. If it is rewarding, then people might show more challenging behaviour in order to obtain deep pressure, in a similar way to the participant in Blairs et al. [10] study who was using challenging behaviour as a way of obtaining restraint and therefore deep pressure.

The study would have been improved if the individuals could have been randomized to different start dates (known as a multiple baseline design [19]). For instance, it could have been organised so that participants began to receive the intervention at weekly intervals, so Alison and Brian might have started on the first week, Carl and David the second week, and so on through the first few weeks of the term. The shift patterns of the staff did not allow this possibility. We would also recommend more systematic measures to ensure reliability and possibly the use of observational measures such as counts of behaviour to evaluate reactions to different techniques of administering deep pressure. Systematisation of observational measures such as those used in this study could include estimation of the reliability of the rating scales and potentially trained raters so as to ensure consistency of measures across raters and occasions. Recent studies of sensory integration have used Goal Attainment Scaling (e.g., [20]) as a method of measuring progress, but other measures such as the Problem Behaviour Inventory [21] might be more relevant when the goal of sensory integration therapy is improved mood or behaviour. Although it is difficult to envisage how this particular group of participants would have reacted to more intrusive measures of response such as galvanic skin response or heart rate monitoring, the development of small wearable monitors offers the potential for still further refinements.

In summary, our study has demonstrated that stimulating skin pressure sensory systems seems to benefit most of a population of young people with autism and severe intellectual disability on whom it was trialled. Importantly, not all participants benefit to the same extent, which suggests that more research is needed to define the populations who might find it most effective.

## Figures and Tables

**Table 1:** It shows the characteristics of the participants. Not all participants had been tested on the psychoeducational profile [14], but all of them had been assessed educationally using the *P* scales as recommended for pupils with special educational needs in England [15].

Participant (number of sessions)	Age (yrs:mths)	Psychoeducational profile [14]						Academic levels	Communication Skills	Reasons for deep pressure
		Cognitive verbal/preverbal	Expressive language	Receptive language	Fine Motor	Gross Motor	VMI			
Alison (130)	07:10	38	26	23	39	29	30	P5	PECS level 3	Banging her head against people
Brian (45)	17:09							P5	PECS level 4	Hitting self, body slamming against walls
Carl (40)	12:10	20	<12	19	23	25	25	P5	PECS level 1	Biting hand
David (17)	13:09	25	21	23	29	27	25	P5	PECS level 4	Dropping to floor
Eric (49)	15:03	26	13	15	23	22	18	P6		Slapping walls
Fenella (17)	14:06	<12	<12	<12	<12	<12	<12	P4	PECS level 4	Dropping to floor
Graham (39)	15:10							P5	PECS level 3	Slapping own head
Herbert (48)	09:05							P4	PECS level 1	Wraps himself in blanket, extremely sensitive to sound

**Table 2:** Means and standard deviations recorded for the scales before and after deep pressure sessions.

Participant (number of sessions)	Scale	Before session mean (SD)	After session mean (SD)	*Z* value of Tau-U statistic
Alison (130)	Calmness^a^	7.84 (2.40)	8.99 (1.59)	6.95^*∗∗*^
	Engaged^a^	7.73 (2.37)	8.87 (1.54)	7.05^*∗∗*^
	Responsivity^a^	8.17 (4.74)	8.88 (1.56)	6.66^*∗∗*^
	Happy	7.26 (2.83)	8.78 (1.62)	5.80^*∗∗*^
	Communicative^a^	7.94 (2.10)	8.87 (1.59)	7.49^*∗∗*^
Brian (45)	Calmness	5.74 (2.51)	7.20 (2.16)	2.96^*∗*^
	Engaged	4.66 (2.40)	6.59 (2.32)	3.65^*∗*^
	Responsivity	4.74 (2.43)	6.39 (2.50)	3.28^*∗∗*^
	Happy	4.92 (2.68)	6.91 (2.19)	3.66^*∗∗*^
	Communicative	5.64 (1.64)	6.78 (1.93)	3.30^*∗*^
Carl (40)	Calmness	7.35 (2.65)	8.84 (1.12)	3.55^*∗*^
	Engaged^a^	6.78 (2.58)	8.51 (1.49)	3.55^*∗∗∗*^
	Responsivity^a^	6.93 (2.56)	8.66 (1.35)	3.72^*∗∗∗*^
	Happy^a^	6.85 (2.83)	8.67 (1.31)	3.71^*∗∗∗*^
	Communicative^a^	5.92 (2.87)	8.08 (2.19)	3.54^*∗∗∗*^
David (17)	Calmness	5.89 (2.49)	8.48 (1.08)	3.29^*∗∗*^
	Engaged	5.52 (2.58)	8.03 (1.57)	2.81^*∗*^
	Responsivity	5.87 (2.44)	8.24 (1.20)	2.91^*∗*^
	Happy	6.15 (2.59)	8.19 (1.53)	2.50
	Communicative	6.22 (2.96)	8.06 (1.78)	1.66
Eric (49)	Calmness	4.68 (2.78)	7.69 (2.04)	4.98^*∗∗*^
	Engaged	4.65 (2.61)	7.27 (2.15)	5.94^*∗∗*^
	Responsivity	5.29 (2.37)	7.65 (2.08)	5.32^*∗∗*^
	Happy^a^	5.69 (2.61)	8.02 (1.86)	3.50^*∗∗*^
	Communicative	5.06 (2.35)	7.55 (1.98)	5.01^*∗∗*^
Fenella (17)	Calmness	5.71 (2.35)	5.99 (2.40)	0.29
	Engaged	4.49 (2.60)	4.90 (2.60)	−0.03
	Responsivity	4.99 (2.36)	4.99 (2.64)	−0.21
	Happy	5.30 (3.01)	5.19 (2.75)	0.26
	Communicative	4.71 (2.76)	4.85 (2.53)	−0.59
Graham (39)	Calmness	7.51 (1.91)	8.66 (1.10)	3.33^*∗∗*^
	Engaged^a^	7.04 (2.41)	8.24 (2.18)	2.20
	Responsivity^a^	7.32 (2.35)	8.31 (1.85)	1.69
	Happy	8.12 (1.86)	9.04 (0.94)	2.56
	Communicative	7.58 (2.14)	8.66 (1.16)	2.85^*∗∗*^
Herbert (48)	Calmness	5.83 (1.91)	6.83 (1.92)	0.11
	Engaged	4.61 (2.13)	6.33 (2.08)	0.12
	Responsivity	5.39 (1.97)	6.40 (1.95)	0.12
	Happy	5.84 (2.13)	6.82 (1.89)	0.11
	Communicative	4.89 (2.07)	5.90 (2.20)	0.12

*Notes.* ^a^Corrected baseline Tau-U statistic used; ^*∗*^*p* < 0.01; ^*∗∗*^*p* < 0.001; ^*∗∗∗*^*p* < 0.0005. Significance figures corrected for multiple imputation per participant using the Bonferroni correction, that is, (0.05/5 = 0.01) used as the highest level of significance reported.

## Additional Points

Deep pressure benefits most participants to a statistically significant degree. The variability in individual responses indicates that deep pressure should be tailored to the individual needs of the recipient. Deep pressure seems to have benefits in many areas for most people, but individual responses must be considered in the design of future research.
